# Structural analysis of eyespots: dynamics of morphogenic signals that govern elemental positions in butterfly wings

**DOI:** 10.1186/1752-0509-6-17

**Published:** 2012-03-13

**Authors:** Joji M Otaki

**Affiliations:** 1 The BCPH Unit of Molecular Physiology, Department of Chemistry, Biology and Marine Science, Faculty of Science, University of the Ryukyus, Nishihara, Okinawa, 903-0213, Japan

**Keywords:** Butterfly wing, Colour-pattern determination, Eyespot, Parafocal element, Induction model, Morphogenic signal

## Abstract

**Background:**

To explain eyespot colour-pattern determination in butterfly wings, the induction model has been discussed based on colour-pattern analyses of various butterfly eyespots. However, a detailed structural analysis of eyespots that can serve as a foundation for future studies is still lacking. In this study, fundamental structural rules related to butterfly eyespots are proposed, and the induction model is elaborated in terms of the possible dynamics of morphogenic signals involved in the development of eyespots and parafocal elements (PFEs) based on colour-pattern analysis of the nymphalid butterfly *Junonia almana*.

**Results:**

In a well-developed eyespot, the inner black core ring is much wider than the outer black ring; this is termed the inside-wide rule. It appears that signals are wider near the focus of the eyespot and become narrower as they expand. Although fundamental signal dynamics are likely to be based on a reaction-diffusion mechanism, they were described well mathematically as a type of simple uniformly decelerated motion in which signals associated with the outer and inner black rings of eyespots and PFEs are released at different time points, durations, intervals, and initial velocities into a two-dimensional field of fundamentally uniform or graded resistance; this produces eyespots and PFEs that are diverse in size and structure. The inside-wide rule, eyespot distortion, structural differences between small and large eyespots, and structural changes in eyespots and PFEs in response to physiological treatments were explained well using mathematical simulations. Natural colour patterns and previous experimental findings that are not easily explained by the conventional gradient model were also explained reasonably well by the formal mathematical simulations performed in this study.

**Conclusions:**

In a mode free from speculative molecular interactions, the present study clarifies fundamental structural rules related to butterfly eyespots, delineates a theoretical basis for the induction model, and proposes a mathematically simple mode of long-range signalling that may reflect developmental mechanisms associated with butterfly eyespots.

## Background

Although butterfly wing patterns are highly complex, it is believed that they are produced by simple rules that determine the fate of immature scale cells fixed in a two-dimensional plane. Among the colour-pattern elements that constitute the overall wing pattern, eyespots are conspicuous symmetric elements. Partly for this reason, characterisation of eyespots via physical damage and transplantation methods has been intensively performed, with the focus on the forewing eyespots of the nymphalid butterflies *Junonia coenia *[1,2] and *Bicyclus anynana *[3-5]. Two other nymphalid butterflies, *Junonia orithya *and *Ypthima argus*, were employed in a similar study [6].

The experimental results obtained in these studies have been explained by the concentration gradient model for positional information, the theoretical basis of which was proposed by Wolpert [7]. Such explanations necessarily exclude alternative models, such as the cascade model, which addresses serial inductive signals, and the wave model, in which signals have an autonomous wave-like character [8,9]. The main reason for this exclusion is the relatively long period of focus dependence in eyespot formation; this phenomenon was revealed when it was observed that focal damage in the early pupal stage resulted in smaller eyespots [1-6]. Following this line of discussion, the putative morphogenic molecules Wingless and TGF-β have been shown to be expressed in at least some eyespots [10]. These molecules are believed to be secreted from prospective eyespot foci and to determine eyespot rings [10-13], although there is currently no functional evidence that the expression of these molecules affects butterfly colour-pattern determination.

Although rarely discussed in the literature, there are several experimental findings and natural colour-pattern variations that have not been explained by the conventional gradient model [14]. Therefore, based on colour-pattern analyses of various nymphalid butterflies, the induction model was proposed as a more realistic alternative [14,15]. In this model, autonomous wave-like signals for dark rings are released from the focus. They are self-enhancing at a short range and induce inhibitory signals at a long range during their expansion and after their settlement, as originally proposed by Gierer and Meinhardt [16-18]. These dark-ring and inhibitory signals may be mutually stabilised and then translated into colour-pattern expression. These processes were simulated computationally using reaction-diffusion equations [15]. The induction model was also shown to be consistent with the results of experimental disruption of eyespots [19].

Nevertheless, there is one important point that has not yet been sufficiently explained by the induction model: how a released wave-like signal "finds" a proper position in which to settle. Because the released wave can progress indefinitely unless it is equipped with a settlement mechanism, this point is directly related to how to organise colour-pattern elements on a wing surface and how to rearrange the nymphalid groundplan in a species-specific fashion.

It is certainly highly likely that the butterfly eyespot system is constructed based on the reaction-diffusion system, and it appears that this settlement problem could be solved by further exploration of the reaction-diffusion model, which could suggest possible molecular interactions. In hydra, the Wnt signal responsible for head development is explained by a reaction-diffusion model [20-23]. The shell colour patterns of molluscs are also simulated by this type of model [24,25]. In both cases, moving signals can be slowed down and become stable under specific conditions. However, these conditions are unlikely to be directly applicable to the butterfly system, considering that in comparison to the hydra system, the butterfly system is much larger, and that in contrast to molluscan systems, it is stable and predictable in a given species, with small individual variations.

It is important to recognise that in any modelling study, the object to be modelled must be understood. That is, structural features of actual butterfly eyespots must be studied first and then the related reaction-diffusion simulations can be explored. Therefore, before approaching to a final solution for the settlement problem, in the present study, I turned my attention to actual colour patterns and did not consider the hypothetical physicochemical nature of signalling molecules included in the gradient and reaction-diffusion models. I do not intend to reject the previously proposed reaction-diffusion mechanism, but I found that more descriptive mechanics are helpful to understand butterfly wings, at least at this point, because of the lack of studies that describe butterfly wing colour patterns in detail. The present study is an attempt to faithfully describe and simulate the observed behaviour of natural and experimentally induced colour patterns using simple equations, without stringently focusing on their hypothetical physicochemical or molecular bases.

**Figure 1:**
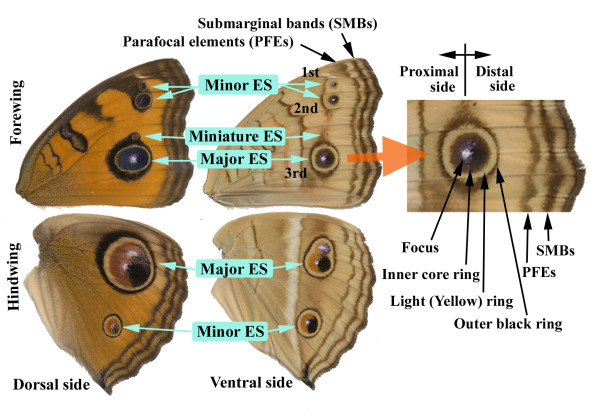
**Wing colour patterns of *Junonia almana***. Each eyespot (ES) is referred to as indicated for convenience. Terms for eyespot substructures and peripheral elements are indicated on the right side.

Throughout this paper, I focus on the eyespots and parafocal elements (PFEs) of the peacock pansy butterfly, *Junonia almana *(Figure 1). The eyespots of this nymphalid butterfly have several notable features that are not found in the eyespots of other butterflies. Specifically, unlike some satyrine butterflies, including *Bicyclus *and *Ypthima*, that exhibit "typical" symmetric eyespots, *J. almana *shows remarkable intra-individual variation in eyespot size and morphology [26,27]. This variation makes morphological comparison between eyespots both possible and fruitful. Based on a colour-pattern analysis of *J. almana*, I present a simple descriptive mathematical model to explain the possible behaviour of morphogenic signals in light of the induction model that is consistent with both observational and experimental results.

## Methods

### Butterflies

The peacock pansy butterfly, *J. almana*, was reared in the laboratory as previously described [27]. Modified eyespots obtained from previous experiments [27,28] were re-examined for colour-pattern analysis of physiologically treated individuals.

### Arrangement and nomenclature of eyespots

Several eyespots are found on the dorsal and ventral sides of the fore- and hindwings of this species (Figure 1). Each wing surface appears to have eyespots organised in a similar configuration. Typically, the dorsal forewing exhibits three eyespots. The major eyespot is located in the compartment CuA_1_, in which the anterior and posterior parts are expanded to the adjacent compartments. Two minor eyespots are found in the compartments M_1 _and R_5_. These major and minor eyespots are distorted toward the wing base. In addition, miniature eyespots are occasionally found in the wing compartments located anterior or posterior to the major eyespot focus. The ventral side presents a similar eyespot arrangement.

The dorsal side of the hindwing exhibits a large eyespot with double foci and a minor eyespot. Both eyespots are distorted toward the wing base. On the ventral side, eyespots are arranged similarly to the dorsal side but are less distorted. The reddish scales on the proximal side of the hindwing core ring are considered to be variants of black scales.

### Basic assumptions

Eyespots (border ocelli) and PFEs together constitute a single system referred to as the border symmetry system [9,28,29]. This means that eyespots and PFEs are determined by the same organising centres. Both of their signals are probably the same or very similar in quality and are released from the same prospective eyespot focus, but at different time points [14,15,27-29]. If the signals are wave-like, then the oscillation of the cellular "medium" can theoretically be characterised by its wavelength and amplitude. However, an oscillation pattern with regular cycles may not be expected; it is more likely that the signals consist of a few sequential trains of progressive peaks of the medium.

The signals involved in eyespot determination primarily specify dark rings, while light rings are passively determined as blank spaces between dark rings [15]. The subsequent modification of the dark and light rings is not considered in this study.

Four steps of elemental formation were considered throughout this report: signalling, reception, interpretation, and expression [27]. The signalling step is relatively long (at least several hours), while the reception step is assumed to be relatively short.

The colour-pattern changes induced by sodium tungstate are equivalent to those caused by cold-shock treatment [30,31], while heat-shock treatment has an opposite effect on wings [26,32]. Tungstate and cold-shock treatments most likely delay the signalling step, while heat-shock treatment accelerates it [28,30]. These physiological treatments are highly important for understanding the formation and positioning of elements because they are the sole experimental means of altering elemental positions, except through physical damage, for the purpose of inferring the signal dynamics in a time sequence.

### Overview of the induction model as a starting point

**Figure 2:**
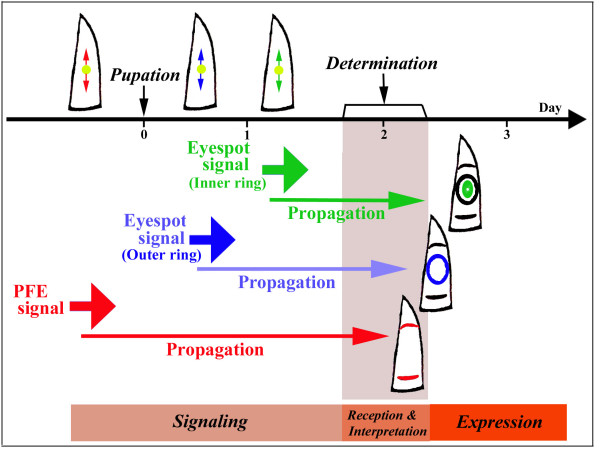
**The time course of signal release and propagation for eyespots and parafocal elements (PFEs) on the dorsal forewing as proposed by the induction model**. The three signals are released at different time points and are propagated autonomously. The mechanisms of signal settlement are unclear in this figure. Note that in this figure, the PFE signal is released before pupation, whereas the eyespot signal is released after pupation. However, this appears to be true only for the forewing; all signals may be released before pupation in the hindwing. Reproduced from Otaki [15]

The induction model is briefly reviewed here as a starting point for the present study (Figure 2) [15]. One of the main characteristics of the induction model is heterochronic signal release. Each dark ring of an eyespot and PFE is determined by an independent signal that is sequentially released at different time points from the prospective eyespot focus. The signal is basically autonomous and mostly independent of the focal activity. Once released, each slowly moving signal settles at a particular position. The signal induces itself at close proximity and induces inhibitory signals at distant positions both while it is moving and after it settles.

Because the PFE signal is released before pupation (Figure 2), the observed PFE behaviours in response to tungstate injection during the pupal stage may only be caused by changes in the signal propagation process. Thus, the changes in PFEs may be an accurate reflection of the signal propagation process. In contrast, the eyespot signal is released after pupation (Figure 2). Thus, eyespot behaviours in response to tungstate injection during the pupal stage may be caused by changes in the activity of organising centres and/or changes in the signal propagation process.

The behaviour of morphogenic signals presented in this paper mainly describes the first (i.e., primary signal expansion and settlement) and not the second (i.e., induction of self-enhancement and inhibitory signals and their stabilising interactions) part of the induction model. In this sense, the first part may be referred to as the rolling-ball model (see mathematical equations discussed below) and the second part as the induction model *sensu stricto*. In reality, these two steps are not separable; however, they are considered separate for the sake of discussion.

### Modes of positional settlement

The possible ways in which propagating signals "find" their settlement positions will be described in the following paragraphs. I propose that the final positional determination may occur via several different modes that are not mutually exclusive.

First, as suggested previously [14,15], the time-out (or end) of the signalling step determines the final pattern because the period of time during which the signal can distribute freely is limited. The signal distribution pattern is captured as a snapshot in a relatively short time based on a quick shift from the signalling step to the reception step, even if signals are still moving slowly. I refer to this as the time-out mechanism of signal settlement. This mechanism is necessary if the pulse-train conditions of the reaction-diffusion equations are operating.

Second, a signal that is released from an organising centre loses its velocity at a particular position before the time-out of the signalling step. This position becomes the base for the final pattern. This mechanism, referred to as the velocity-loss mechanism of signal settlement, can be divided into two modes. One mode is simply caused by a low-level signal that cannot expand over a far distance and, thus, settles when it loses velocity. In other words, the initial velocity of a signal and the deceleration rate in the running medium determine how far the signal runs. In the case of relatively small eyespots, this mode may be the major positional determinant because its signal level from the organising centre is inherently low; I refer to this as the spontaneous velocity-loss mechanism of signal settlement.

In the other mode of the velocity-loss mechanism, an expanding signal is repulsed by another signal located nearby and, thus, loses velocity; I refer to this as the repulsive velocity-loss mechanism. Because the dark ring signal not only enhances itself but also induces an inhibitory signal [15], the two elements can repulse each other, even though they do not appear to have any physical contact. The front-most inhibitory signals of eyespots occasionally form "imaginary rings" that often deform nearby PFEs [15].

These different modes are not mutually exclusive. Indeed, they may coexist on a single wing surface. However, it is important to stress that the time-out mechanism does not achieve a steady state of signal distribution, whereas the velocity-loss mechanism (in both spontaneous and repulsive modes) can establish a steady state if the time span of the signalling step is sufficiently long. A reasonable theory concerning signal settlement is that although the time-out mechanism is perfectly allowable, signals are equipped with velocity-loss mechanisms. Otherwise, independent evolution of each element on a single wing surface would be very difficult, and highly diverse nymphalid colour patterns would not be possible. In the following sections, I examine the colour patterns of *J. almana *in light of these modes of final positional determination.

## Results

### Colour-pattern analysis of eyespots and PFEs in *J. almana*

#### The inside-wide rule for eyespot rings

The major forewing eyespot on the ventral side of *J. almana *exhibits a relatively symmetric circular pattern (Figure 1). One eyespot contains two black rings: the inner core ring and the outer ring. The inner core ring is much wider than the outer ring. Similar tendencies are observed in well-developed eyespots located on all wing surfaces in this species and others. I refer to this universally observed morphological feature of butterfly eyespots as the "inside-wide rule," although there are some important exceptions to this rule, which will be discussed later.

It might be concluded that the inside-wide rule suggests that the outer signal is weaker (or released for a shorter period of time) than the inner signal from the beginning of signal release. An alternative view is that these two signals are not very different in their signal intensity initially; however, the signals become narrower as they expand. In other words, the outer ring is narrower simply because it travels farther. This latter possibility is more likely because PFEs behave this way in response to tungstate treatment.

Based on the discussion presented above, the dynamics of the morphogenic signals that generate the inside-wide configuration of eyespots potentially occur as follows. First, the signal for the outer black ring is released; second, the signal for the inner core ring is released after a relatively long pause. The first signal becomes sharper as it expands due to deceleration and settles at a particular position. The inner signal then gradually catches up with the outer signal, causing the light ring to be narrower. Thus, the time-out or velocity-loss mechanism (including both spontaneous and repulsive modes) can produce the inside-wide eyespots.

#### Eyespot size and structural differences

**Figure 3:**
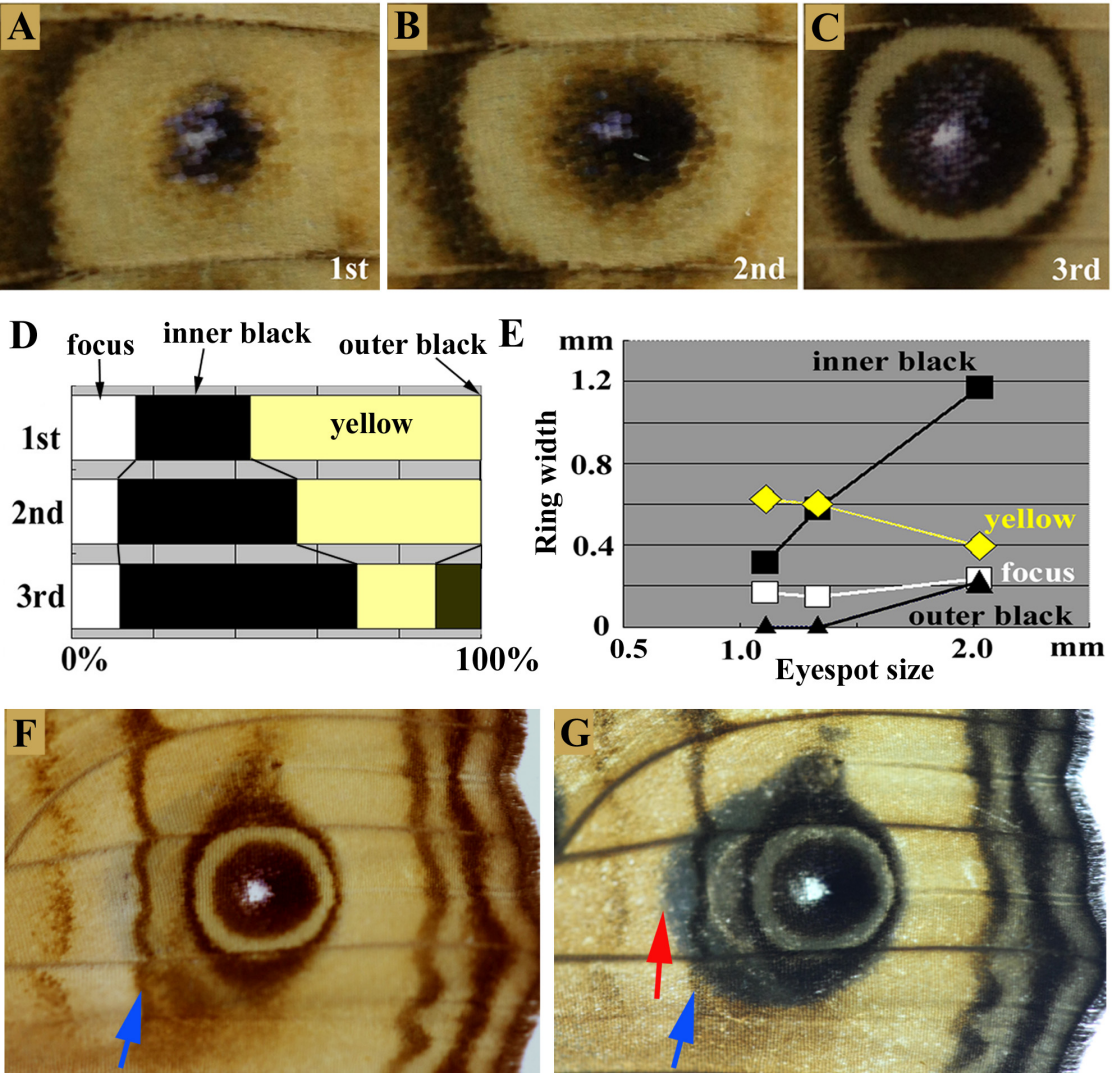
**Colour-pattern analysis of eyespots on the ventral forewing**. (A-C) There are three eyespots on the same wing surface: 1st (minor), 2nd (minor), and 3rd (major). The eyespots are enlarged to similar sizes in these photographs. Refer to Figure 1 for their relationship on the wing. Note the differences in the ring width proportions. Modified from Otaki [14]. (D) Ring width ratios in the three eyespots shown in A-C. Small eyespots have wider yellow-ring width proportions. Modified from Otaki [14]. (E) Relationship between eyespot size and ring width. As the eyespot size becomes larger, the inner black ring becomes wider and the yellow ring narrower. Modified from Otaki [14]. (F) The major (3rd) eyespot and other nearby elemental positions on the ventral forewing. The blue arrow indicates the element that could block the propagation of the eyespot signal proximally. (G) A wing identical to that shown in F; however, the wing is illuminated from the bottom upward so that both the dorsal and ventral colour patterns can be simultaneously observed. The blue arrow indicates the position identical to that shown in F. The red arrow indicates the edge of the outer black ring on the dorsal side.

The inside-wide rule applies almost universally to the well-developed eyespots of nymphalid butterflies, though small or immature eyespots (*sensu *Otaki [15]) represent exceptions to this rule. The ring widths of three eyespots of different sizes on the ventral forewings were examined for the purpose of discussing this point more quantitatively. The widths of the inner core rings clearly increased in relation to eyespot size (Figure 3A-E) [15], which likely caused the width of the light rings to decrease in relation to eyespot size. These structural differences among eyespots of different sizes may be mainly due to differences in focal activity [4]. Therefore, the spontaneous velocity-loss mechanism mainly explains the formation of small or immature eyespots.

In contrast, on the dorsal side of the forewing, the width of the light rings did not vary considerably between the major and minor eyespots (Figure 1). The induction model explains the relatively constant light rings as follows. On the dorsal side, the outer and inner signals are released without a considerable delay, and the inner signal soon catches up with the outer signal; an inhibitory signal released between the two signals prevents them from fusing with each other. The narrow and less variable light rings on the dorsal side are therefore an expression of the minimum possible gap width in between which the outer and inner rings can form.

#### Elemental repulsion

Assuming that the signal intensity of the major eyespot foci on the dorsal and ventral sides of the forewing is similar, the reason for the anatomical differences between the dorsal and ventral sides requires a logical explanation. A transparent image of the dorsal and ventral sides readily revealed that the dorsal eyespot was elongated on the proximal side, whereas the ventral eyespot was compressed (Figure 3F, G). In contrast, the dorsal and ventral eyespots were nearly identical in size on the distal side, suggesting that their focal activities were similar. Notably, there was a line-like element located in the basal region of the ventral side that was not found in the basal region of the dorsal side (Figure 3F, G). This anatomical difference could partially explain why the major eyespots on the dorsal sides are much larger than those on the ventral sides in the proximal-distal axis (more specifically, only on the proximal side), but not in the anterior-posterior axis. The underlying mechanism for this pattern could be repulsion between elements that might be mediated by the outermost imaginary rings of the inhibitory signals. Therefore, the repulsive velocity-loss mechanism at least partially explains this eyespot distortion.

In the literature, eyespot distortion has been explained by the two-gradient model [8,33,34]. The existence of a wing-wide gradient is acceptable both in the conventional gradient model and in the induction model.

#### Elemental fusion

A double focal eyespot was found on the dorsal and ventral sides of the hindwing (Figure 1). A double focal eyespot is the product of two eyespots fused together at an earlier stage of signal development: two signals are fused instead of repulsed. Fusion may occur if the inhibitory signals have not developed sufficiently to repulse one another. Fusion is usually observed between closely located eyespots, which could indicate that they were in contact with each other at a relatively early stage of signal development when the inhibitory signal was not strongly induced.

#### Physiologically induced changes in eyespots

**Figure 4:**
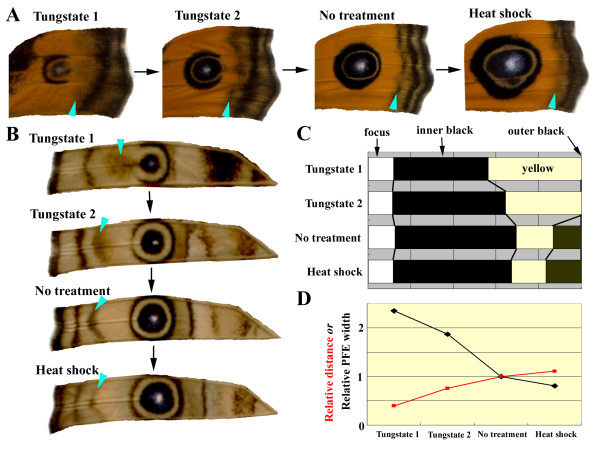
**Colour-pattern analysis of tungstate-induced and heat-shock-induced modifications of major eyespots on dorsal and ventral surfaces**. (A, B) Dorsal (A) and ventral (B) major eyespots and PFEs of the forewing. Four individuals were aligned according to the possible time sequence of development. Treatment modes are indicated above each wing portion; light blue arrowheads indicate PFEs. Smaller eyespots and PFEs that were dislocated closer to the eyespot focus were considered to represent earlier stages of development. The open area produced by tungstate treatment on the distal sides of smaller eyespots may be attributed to repulsion from PFEs located nearby. Note the size and structural changes of the eyespots. Additionally, note the positional and width changes in the PFEs. Modified from Otaki [27]. (C) Percentages of white foci, inner black core rings, yellow rings, and outer black rings of the major eyespots in the four individuals shown in B. The distal sides of the major eyespots were measured from the centres of the focal areas. (D) Relative distances of PFEs from the focus and relative PFE widths in the four individuals shown in B. The distance was measured from the centre of the focus to the nearest part of the PFEs. The distance and width of the "no treatment" individual were adjusted to 1.00, and other data were normalised accordingly.

The eyespots in an individual butterfly that received a tungstate injection were smaller than those in non-treated individuals (Figure 4). The inner orange ring of the modified dorsal major eyespot was wider than that of a normal eyespot based on the proportion of the size of the whole eyespot (Figure 4A). Similar results were obtained for the modified ventral major eyespot (Figure 4B). These modified major eyespots exhibited a similar ring structure to the normal minor eyespots on the same wing surface (compare Figures 4C and 3D).

It is likely that the tungstate treatment revealed earlier developmental time points involved in normal eyespot development by delaying the signalling step [27,29]. Thus, it is possible to infer that there is a relatively long pause between the release of the outer black signal and the inner core signal in an earlier stage of eyespot development, even on the dorsal side. Subsequently, the inner core signal soon catches up with the outer signal. Furthermore, there may be an inhibitory signal between the PFE and the eyespot outer ring that causes the disappearance of the distal sides of the modified eyespots.

#### Physiologically induced changes in PFEs

Structural changes in the forewing PFEs induced by the physiological treatments revealed that the closer to the eyespot focus a PFE was located, the larger it became (Figure 4D). The tungstate and cold-shock treatments likely decrease the initial velocity of the signal, which then shortens the propagation time (or decreases the propagation speed) [27,29]. It can be concluded that a PFE signal released for a defined time span settles by gradually decreasing its velocity.

However, heat-shock treatment can further dislocate PFEs toward the wing margin. Moreover, submarginal bands (SMBs) located near the PFEs are dislocated together with the PFEs in the same directions. These results suggest that the PFE signal has the velocity to proceed farther toward the wing margin and that the repulsive velocity-loss mechanism of signal settlement may be operating between the PFEs and the SMBs. Similar dynamics could also be applicable to eyespots.

### Mathematical model

#### Uniformly decelerated motion

Ignoring physicochemical mechanisms, the signal dynamics discussed above can be mathematically modelled based on simple uniformly decelerated motion, which is a special case of uniformly accelerated motion, but with a negative acceleration rate (i.e., a deceleration rate). It is possible to hypothesise that the medium for signal propagation presents a significant degree of resistance that is uniform throughout a given wing surface. This resistance determines the deceleration rate of the signals.

Classical mechanics states that a given position *x *of a linearly moving object (i.e., a morphogenic signal in this case) in a medium with uniform resistance from the original position with a constant negative acceleration rate *a *(< 0) (i.e., a deceleration rate) and initial velocity of the signal *v_o _*is expressed as a function of time (*t*) as follows:

(1) $$x=v_{0}t+\left(1/2\right)at^{2}$$

That is, *x *is expressed as a quadratic equation of *t *with real coefficients. In an ideal situation, *v_0 _*and *a *are fixed for a given signal. The maximum value of *t *can be obtained when *dx/dt *= 0 as follows:

(2) $$dx/dt=v_{0}+at=0\Rightarrow \;t=-v_{0}/a$$

Simply because *a *is negative, a released signal stops when its velocity becomes zero. Hence, the effective ranges of *t *and *x *in Eq. (1) are shown below:

(3) $$0\leq t\leq -v_{0}/a;\;0\leq x\leq -v_{0}^{2}/2a$$

That is, the maximum value of *x *(i.e., the final position of a signal) is given by-*v_0_*^2^/2*a *at time point-*v_0 _a*. When *t *exceeds this point, *x *becomes constant, as follows:

(4) $$x=-v_{0}^{2}/2a\;\;\left(t\geq -v_{0}/a\right)$$

Suppose that the negative acceleration rate *a *is uniform on a given wing surface, for example, *a *= -1. Then, Eq. (1) becomes simpler, as follows:

(5) $$x=v_{0}t-\left(1/2\right)t^{2}$$

Additionally, the ranges of *t *and *x *can be expressed as shown below:

(6) $$0\leq t\leq v_{0};\;0\leq x\leq v_{0}^{2}/2$$

That is, when resistance is uniform, the location of a signal is dependent on time and initial velocity. The maximum value of *x *is given by *v_0_*^2^/2 at time point *v_0_*. When *t *exceeds this point, *x *becomes constant as follows:

(7) $$x=v_{0}^{2}/2\;\;\left(t\geq v_{0}\right)$$

**Figure 5:**
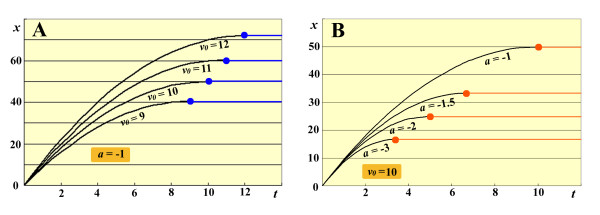
**Signal dynamics according to Eq. (1)**. The signal position *x *changes as a function of time *t*. (A) *t*-*x *curves when *a *= -1 with various values of *v_0 _*according to Eq. (5). Blue dots indicate the maximum (final) positions for each value of *v_0_*. Blue lines indicate the constant positions after the blue dots. (B) *t*-*x *curves when *v_0 _*= 10 with various values of *a *according to Eq. (8). Orange dots indicate the maximum (final) positions for each *a*. Orange lines indicate the constant positions after the orange dots.

For example, consider Eq. (5) with *v_0 _*ranging from 9 to 12 for the sake of simplicity. Depending on *v_0_*, the position *x *varies as shown in the *t*-*x *plot (Figure 5A), indicating that the initial velocity *v_0 _*is an important factor for determination of eyespot size.

Now, suppose that the initial velocity, *v_0_*, is uniform on a given surface and that the negative acceleration rate *a *is variable. Acceleration may indeed vary depending on wing surfaces and on other elements located nearby due to the repulsive velocity-loss mechanism. For example, if *v_0 _*= 10, then Eq. (1) is as follows:

(8) $$x=10\;t+\left(1/2\right)at^{2}$$

A *t-x *plot of Eq. (8) using various *a *values shows that increasing the absolute value of *a *causes the final position to become nearer to the focus (Figure 5B), indicating that the acceleration rate *a *is also an important factor for determination of eyespot size. Because *a *is distributed in a two-dimensional plane, different (or graded) values of *a *at different positions could influence the final eyespot morphology.

#### Signal duration, interval, and other structural determinants

Although a signal may follow Eq. (1), it is not sufficient to depict an eyespot. A given eyespot dark ring has a certain width, which means that a single signal does not occur in the form of a sharp pulse but is more likely to be released for a certain time period. Thus, the signal duration *D *is also a structural determinant. A dark-ring signal may be considered to be composed of minute unit signals, such that every unit shows identical behaviour, but with a slight time difference. The velocity of the signal front declines first, and that of signal rear declines last. Therefore, as the band of signal travels farther, its width becomes narrower.

In addition, the signal interval, *I*, which is the difference between the released time points (the end point of the outer ring signal minus the initial point of the inner ring signal), is another determinant that is required to construct a typical eyespot with two dark rings.

In summary, determinants of eyespot structure include the signal duration *D *and the signal interval *I *in addition to the negative acceleration rate *a *and the initial velocity *v_0_*. The number of signals *n *(or the number of cycles) may also be considered as a determinant, but this number is usually 2 (for outer and inner rings). Of course, if the time-out mechanism of signal settling is operating, the duration of the signalling step, or the maximum *t*, is another determinant. The efficiency of inhibitory signal induction during the second step of the induction model may also contribute to the final structure. However, this aspect of the induction model is beyond the scope of the present study.

#### Simulations of "typical" eyespots

**Figure 6:**
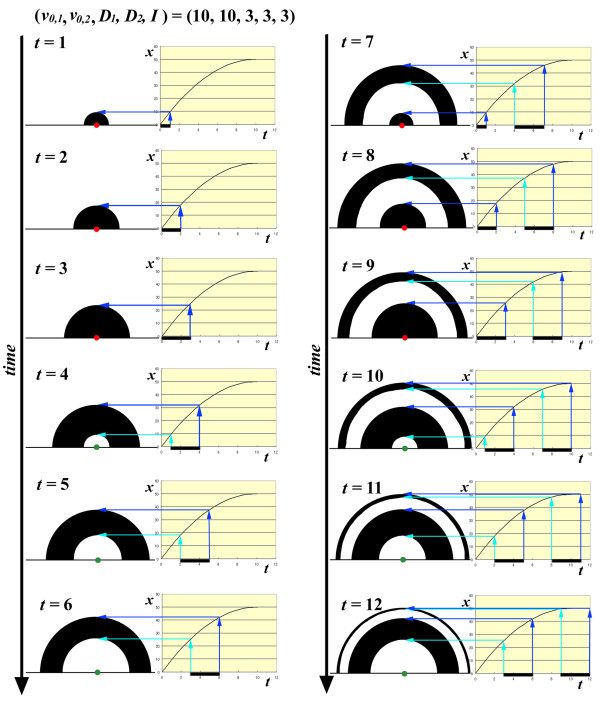
**Simulation of eyespot signal expansion with a fixed initial velocity *v_0_***. Two signals (*n *= 2) with the same initial velocity (*v_0 _*= 10) and signal duration (*D *= 3) were assumed. The signal interval *I *was set at 3. The released signals are distributed in a two-dimensional plane based on the *t-x *curve shown on the right side of each column. Signal durations are indicated by horizontal bars under the *t *axis. The signal front is indicated by a blue arrow and the signal rear by a blue-green arrow. Only half of an eyespot is drawn. Red focal dots indicate active organising centres releasing the signal, whereas blue dots indicate organising centres pausing during the signal intervals. As time progresses from *t *= 1 to *t *= 12, the widths of both black rings and light rings change dynamically. Under these conditions, typical eyespots probably lie within 8 ≤ *t *≤ 10

This section discusses how the above mathematical and conceptual descriptions of signal dynamics can produce an eyespot. For simplicity, suppose that two signals are released from an identical organiser (*n *= 2) under the following conditions for both signals: *a *= -1; *v_0 _*= 10; *D *= 3 for both signals; and *I *= 3 (Figure 6). As a function of time, the signal distribution patterns produce various eyespots. Under these conditions, "typical" inside-wide eyespots were depicted at *t *= 9 and 10. The time-out mechanism or repulsive velocity-loss mechanism is necessary for these eyespots to be fixed in a typical shape.

These factors can be adjusted so that more diverse eyespot patterns are produced, such as under conditions where two signals have different initial velocity values. In all cases, the signals become sharper as they travel farther because the released signals for a given period of time converge on the identical position if they are allowed to travel until they completely lose the velocity to proceed.

#### Simulations of small eyespots

Small eyespots on the ventral forewing were simulated. These eyespots could be naturally small (i.e., minor eyespots), or their small size could be a result of physiological treatments. As discussed above, weak activity of an organising centre would have two consequences: a low initial velocity and a shorter signal duration. The outer signal of the small eyespots would be located almost at the final position, making the outer ring very narrow. In this case, the spontaneous velocity-loss mechanism of signal settlement would be operating.

**Figure 7:**
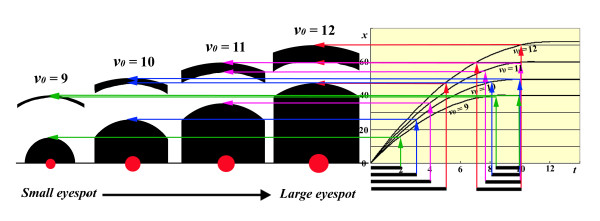
**Simulation of eyespots with various levels of organising centre activity**. Signals propagate according to the *t-x *curves depicted on the right side of the figure. By definition, the signalling process starts at *t *= 0 for all four eyespots shown in this figure. However, smaller eyespots were assumed to be associated with a lower *v_0 _*and shorter signal duration *D*. The signalling dynamics shown in Figure 6 are not shown here, but the single time point *t *= 10 is depicted as a snapshot. Signal durations are indicated by horizontal bars under the *t *axis. The front and rear of the signal for the same eyespot are depicted by arrows of the same colour. Organising centres are shown as red spots; the sizes of the spots reflect their signalling activities. Only half of an eyespot is drawn, with both the right and left sides deleted for simplicity. Note the differences in size and morphology among these four eyespots.

Here, four eyespots with different sizes were depicted at *t *= 10 (Figure 7). By definition, the prospective foci initiate the signalling step at *t *= 0 (which does not mean that they actually initiate the signalling step simultaneously on a wing), but their initial velocity *v_0 _*and signalling duration *D *are different from one another. Small eyespots are associated with a low initial velocity and a shorter duration of signalling. Furthermore, for small eyespots, there are a longer intervals before the beginning of the second signal. That is, the release of the second signal is delayed for smaller eyespots. Based on these reasonable conditions, the structural features of the small eyespots indicated in Figures 3 and 4 were successfully reproduced.

#### Simulations of physiologically modified PFEs

**Figure 8:**
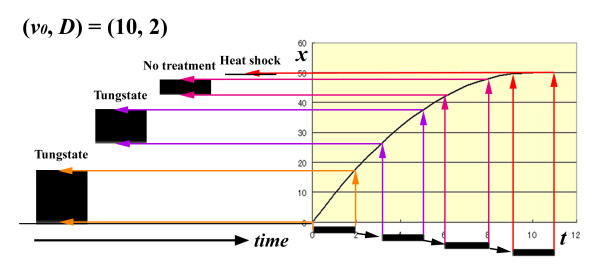
**Simulation of PFE signal expansion**. The initial velocity, deceleration rate, and signal duration are fixed at *v_0 _*= 10, *a *= -1, and *D *= 2, respectively. Signals are propagated according to the *t-x *plot shown on the right side of this figure. Signal durations are depicted by horizontal bars under the *t *axis. The front and rear of a signal for a single PFE are indicated by arrows of the same colour. As time progresses, the position and size of the PFEs change. Treatments that produced similar PFEs in Figure 4 are indicated above each simulated PFE.

Similar to the results for the eyespots, PFE behaviour in response to tungstate and temperature treatments (Figure 4) was properly simulated (Figure 8). In the induction model, the PFE signal has already been released at the treatment time point. Therefore, the initial velocity is not affected by the treatment. In contrast, the propagation time (or speed) of the released signal is affected. Consistent with the experimental changes, PFEs located closer to the focus are wider in this simulation.

### Natural and experimentally induced colour patterns that are not explained by the gradient model

#### Variations in natural colour patterns

The previously proposed induction model [14,15,19] is strengthened by the settlement mechanisms discussed above. For this model to be valid, it should be able to explain most experimental findings and the wide variety of natural colour patterns, which the conventional gradient model is unable to explain, despite its popularity.

Otaki [14] discussed several findings that cannot be explained by the conventional gradient model. First, miniature and minor eyespots usually exhibit different ring widths compared to major eyespots on the same wing surface. This difference is not consistent with what is predicted by the gradient model. As discussed in this report, the induction model explains this difference based on variations in the initial velocity and signalling duration.

Second, ring-dependent distortion is observed in many eyespots. This distortion is perplexing from the viewpoint of the gradient model [14]. In the induction model, ring-dependent distortion is at least partially solved by the fundamental assumption that the signal is related only to dark (usually black) rings and not to light rings. For example, when the proximal side of two dark rings of an identical eyespot is distorted toward the wing base, it is logical that the light ring between the dark rings will appear to be distorted toward the opposite direction because the light ring is a blank space between the dark rings.

Third, several types of "atypical eyespots" that cannot be logically explained by the gradient model, such as an eyespot without an inner core-ring area (blank-core eyespot), a focus-only "eyespot," an eyespot with multiple or scattered foci, and a multi-ring eyespot, can be explained via activity changes in organising centres. A blank-core eyespot readily arises when the inner black signal is not released after the release of the outer black signal. Similarly, the lack of an eyespot with a PFE in a given wing compartment can be understood as the cessation of organising activity after the release of the PFE signal. Focus-only eyespots likely occur because of the very low initial velocity of a signal that was nevertheless able to delimit the focal area. An eyespot with multiple or scattered foci can be produced if the small signals from multiple foci are additively integrated to signify the outer rings. Multiple rings (three or more dark rings) in an eyespot are readily explained based on the oscillatory activity of focal cells that release signals three times or more (*n *≥ 3).

#### Width of light rings in graft-induced eyespots

French and Brakefield [4] experimentally determined that graft-induced eyespots were always smaller than normal eyespots and contained significantly wider yellow rings. The general size reduction observed for graft-induced eyespots may be explained by the shorter time period that is available for releasing the signal from the grafted organising centres. However, there was no logical explanation given for the wider yellow ring.

The smaller graft-induced eyespots with a wider yellow ring are quite reminiscent of naturally occurring minor eyespots and small tungstate-induced eyespots. Using this morphological analogy, it is likely that the grafting process weakened the organising centre. More specifically, the grafting process reduced the initial velocity of the signal, shortened the signal duration, and lengthened the signal interval due to the general decline in the activity of the grafted organising centre.

#### The hindwing paradox

Nijhout [8] demonstrated that hindwing eyespots could not be reduced in size by focal damage in *J. coenia*. Nevertheless, considering that damage to the non-eyespot area of hindwings at the pupal stage produces ectopic eyespots and that temperature shock at the pupal stage can alter the size and shape of hindwing eyespots, it appears that scale cell fate is not yet determined at the time of damage [8]. These results are quite different from what is observed for the forewings and, thus, cannot readily support the gradient model. I refer to these seemingly perplexing results the "hindwing paradox."

Nijhout [8] explained the hindwing paradox by introducing the morphogen sink hypothesis, which states that eyespots on the dorsal forewings are produced around a morphogen "source," whereas eyespots on the dorsal hindwings are produced around a morphogen "sink." Thus, non-focal damage to hindwings creates an artificial sink that then produces an ectopic eyespot. A similar model was proposed for *B. anynana *in which non-focal damage to dorsal forewings creates a morphogen sink and, consequently, an ectopic eyespot [3-5]. Furthermore, a similar model was proposed for black spot formation in the cabbage white butterfly *Pieris rapae *[35,36].

However, it is difficult to believe that morphologically homologous structures on the fore- and hindwings of an individual are determined by opposite mechanisms (i.e., sources vs. sinks). Why damage at the sink (i.e., at the hindwing eyespot focus) cannot halt the focal sink-producing activity on hindwings is not entirely clear. Furthermore, it is difficult to believe that non-focal damage to forewings produces a morphogen source, whereas non-focal damage to hindwings produces a morphogen sink in *J. coenia *(and not necessarily so in other species). In addition, there is no qualitative difference between the fore- and hindwings observed in response to pharmacological and temperature treatments [26,27,30-32], although the opposite effects of these treatments might be expected in light of the morphogen sink hypothesis. Finally, at least in *J. almana*, focal damage to the hindwing can successfully reduce eyespot size (Otaki, unpublished data).

The induction model can readily explain the hindwing paradox. Hindwing eyespot signals have already been released and are running during the early pupal stage according to Eq. (1), which makes them insensitive to focal damage. However, the signalling step continues at this stage, so ectopic signals can be accommodated. In contrast, focal damage to the forewing reduces the number of functional cells before or during the release of the signal. Therefore, the hindwing paradox can be explained by the difference in the timing of signal release between the fore- and hindwings. This explanation is consistent with the results of physiological treatments in which the colour patterns of the dorsal forewings are likely determined last among the four wing surfaces [29].

I have previously discussed the inconsistent behaviours of PFEs in response to experimental damage and physiological treatments from the viewpoint of the conventional gradient model, which has been referred to as the "PFE paradox" [28]. Because the dark rings of eyespots are developmentally equivalent to PFEs [28,29], the hindwing paradox is essentially equivalent to the PFE paradox.

## Discussion

### Status of the induction model

Even if the induction model can explain most cases of butterfly wing colour patterns, one big question remains: is the induction model actually at work in real wings? This question arises because long-range signalling for developmental determination that travels according to a simple, uniformly decelerated type of motion is currently unknown in developmental biology.

I admit that uniformly decelerated motion may merely be an approximate behaviour. One could further argue that the success of mathematical simulations may be a pure coincidence. Classical mechanical equations are, after all, descriptive empirical equations formulated without consideration of the fundamental interactions between macroscopic objects. Eventually, the butterfly system may be described entirely by reaction-diffusion models that are more precise than those proposed previously [15]. Nevertheless, the fact that the formal model presented in this paper can explain diverse natural and experimentally induced eyespot patterns indicates that uniformly decelerated motion represents a reasonable approximation at this point, despite the fact that no molecular mechanism can be suggested by this model.

Currently, the induction model does not consider any molecular interaction imposed by the physicochemical nature of biological molecules. Rather, the induction model is fundamentally based on colour-pattern analysis of diverse butterfly wings. This approach contrasts with the conventional gradient model, which was proposed based on speculative molecular properties of a putative morphogen and its receptor but paid virtually no attention to actual butterfly colour-pattern diversity. Therefore, a molecular model based on the reaction-diffusion system that does not conflict with this formal model is to be proposed in the future. In addition, the results of a recent report on artificially induced colour patterns in *J. almana *[19] may have to be incorporated within the framework of these mathematical simulations.

### Possible molecular and cellular mechanisms of signal propagation

The mathematical model described in this paper is analogous to a ball rolling on a plane. In the biochemical dimension, "rolling a ball (i.e., a molecule)" in a system appears to be rather difficult, if not impossible, due to the thermal motion of the "ball" and other surrounding molecules as well as the viscosity imposed by cellular environment (i.e., under the conditions of low Reynolds number). The ball (i.e., the signal itself) would not be a moving physical object but, rather, would be the moving pattern of a "medium" (i.e., a wave).

A somewhat similar system is observed in the propagation of action potentials in neurons, in which a short refractory period prevents backward signal propagation. However, in the case of butterfly wings, there would be no excitatory membrane potential based on voltage-gated channels in immature scale cells; even if this potential exists, the positive feedback mechanism involved in action potential propagation is inconsistent with the attenuating nature of the signal required in the induction model. The propagation of receptor potentials in sensory neurons may be more analogous to a rolling ball. Similarly, a calcium wave [37], a gene expression wave [38], or other types of cell-to-cell interactions may be consistent with a rolling ball model. The time scale of signal propagation, which is on the order of hours in butterfly development, is more consistent with a gene expression wave, although it would be possible for rapid repetitive electrical or calcium waves to induce a slow expression wave. Unfortunately, it is currently difficult to evaluate the functionality of candidate signalling molecules expressed during eyespot development. However, the putative involvement of the Wingless/TGF-β signalling system in butterfly eyespot development [10] indicates an analogy to the reaction-diffusion mechanism found in hydra [20-23].

Decelerated motion means that kinetic energy is provided at the beginning of motion and is not supplied as a signal moves linearly. That is, only the initial velocity contributes to the kinetic energy of a rolling ball, and the kinetic energy is gradually converted to thermal energy. Creating such directional motion in a sea of random thermal motion at the molecular level without an outside energy supply would appear to be a violation of the second law of thermodynamics because work (or order) is created from an isothermal state. A more realistic alternative is that there is a gradual decline in the rate of energy supplied to the moving signal.

It has been argued that Maxwell's demon, which can "watch" random molecular motion and "select" particular molecules, could achieve similar results without violating the second law of thermodynamics. The experimental existence of Maxwell's demon (i.e., the feasibility of such a system without violation of the second law) has been recently demonstrated [39]. Therefore, it is at least theoretically possible that order is created by extracting information from thermal motion. It would be highly interesting if the butterfly colour-pattern system were to be found to take advantage of Maxwell's demon. Notably, similar mechanisms may be involved in some protein dynamics [40].

### Colour patterns not explained by the induction model

Although the induction model is widely applicable to various types of eyespots, it is far from perfect. There is one notable phenomenon that the induction model cannot explain: the existence of a core ring containing two colours, as observed in the hindwing eyespots of *J. almana*. In the case of *J. almana*, the proximal side of the core ring is reddish, whereas the distal side is not (Figure 1).

The reddish coloration is probably produced in the interpretation and expression steps similarly to a light-ring modification process [15]. The developmental mechanism underlying the dual coloration of core rings is therefore beyond the scope of the induction model. In the future, its relationship to the induction model needs to be clarified.

## Conclusions

In this paper, I described the possible dynamics of morphogenic signals that determine butterfly wing colour patterns based on the inside-wide rule and other structural features of *J. almana *eyespots. Overall, the induction model, which is conceptually developed based on colour-pattern comparisons and includes descriptive mathematical equations, can explain not only the diverse morphology of eyespots and PFEs but also experimental findings that have previously been enigmatic. Although its physicochemical and molecular basis is entirely unknown, this type of long-range molecular signalling mechanism employed in butterfly wings may be generally applicable to other biological systems.

## Competing interests

The author declares that he has no competing interests.
